# Temporal and Spatial Changes in Black Carbon Sedimentary Processes in Wetlands of Songnen Plain, Northeast of China

**DOI:** 10.1371/journal.pone.0140834

**Published:** 2015-10-15

**Authors:** Jiabao He, Chuanyu Gao, Qianxin Lin, Shaoqing Zhang, Winston Zhao, Xianguo Lu, Guoping Wang

**Affiliations:** 1 Key Laboratory of Wetland Ecology and Environment, Northeast Institute of Geography and Agroecology, Chinese Academy of Sciences, Changchun, China; 2 University of Chinese Academy of Sciences, Beijing, China; 3 Department of Oceanography and Coastal Sciences, School of the Coast & Environment, Louisiana State University, Baton Rouge, Louisiana, United States of America; 4 Smeal College, Penn State University, University Park, Pennsylvania, United States of America; Shandong University, CHINA

## Abstract

Black carbon (BC), an important component of organic carbon (OC) produced from incomplete combustion of carbon compounds, is widespread and affects the global carbon storage. The objectives of this study were to analyze the BC contents and fluxes in the last 150 years to determine the causes of differences in the three profiles of the Songnen Plain of Northeast China and to estimate the BC storage in the wetlands of the Songnen Plain. In the three sampling sites, BC fluxes in the period between 1950 and the present time increased by the ratios of 1.3, 31.1 and 1.4, respectively, compared to their own baseline between 1850 and 1900. Furthermore, the BC fluxes varying from 0.76 to 5.63 g m^-2^ y^-1^ in the three profiles had an opposite trend with the sand percentages with mean values changing from 78.9% to 19.6%, suggesting that sand desertification might additionally affect the BC processes in the region.

## Introduction

Black carbon (BC), chemical carbon compounds formed with incomplete combustion of biomass or fossil fuels [1], is an important component of organic carbon (OC) [2]. BC produced during combustion could emit fumes into the atmosphere as a form of aerosol that affects the climate [3, 4] and Earth’s radiative heat balance [5]. BC could reach remote areas via a long-range atmospheric transport, deposit on the earth’s surface by dry or wet precipitation [6], and thus distribute over the earth extensively (e.g. icecaps, peatlands, and lake sediments) [7–9]. BC has been used as indicators to study the melting of glaciers, the history of fire activity, and the estimation of carbon sequestration potential in soils [8, 10, 11]. The global BC production was estimated to be in a range from 4×10^13^ to 60×10^13^ g per year [12], whereas the reservoir of BC in global scale has been estimated at 3–5×10^17^ g [13]. It was estimated that 1.6×10^18^ g of carbon in the soil pool has been stored, of which 28.4% are stored in wetlands [14]. Although lack of a universal method to estimate BC storage in wetlands, especially in peatlands, could result in large variation.

A number of factors could influence BC contents and fluxes. Human activities and environmental changes could be primary driving forces influencing BC contents and fluxes. Gao et al. (2014) [7] indicated that human activities were the main BC source in the region of Sanjiang Plain (Northeast of China), and caused dramatic increase in BC between 1950s and 1980s. The study of Lake Qinghai (Northwest of China) by Han et al. (2015) [15] additionally indicated the influence of human activities on ratios of char and soot in the northern sub-basin in 1980s. Furthermore, a number of studies have shown that the trend of BC coincided with the changes of ecosystems, such as burning, droughts and monsoon changes [16–19].

The Songnen Plain was formed by alluvial deposits [20]. In addition to semiarid conditions, the region has been greatly influenced by the process of sand desertification like most areas in northern China [21]. From the late 1950s to the early 2000s, the rate of sand desertification has increased from 1560 km^2^ to 3600 km^2^ annually [22]. The relationship between OC and sand or clay has been reported. Su and Zhao (2003) [23] indicated that in Horqin sandy land (West of China), SOC and N associated with soil particles substantially decreased with an increase in desertification. A study on the cold semiarid grasslands of Qinghai-Tibet Plateau (Northwest of China) showed that from slight to very severe stages of desertification, the proportion of clay decreased by about 30%, while sand increased by 76%. As a result, SOM and nutrients reduced and the pattern of vegetation changed with desertification process [24]. Although there have been a number of studies on OC and desertification, the variation and storage of BC with the process of sand desertification is not well understood, especially in wetlands. The objectives of this study were (1) to quantify the BC contents and deposition fluxes of three profiles in the Songnen Plain, (2) to analyze the potential influences of human activities and sand desertification on BC deposition fluxes, and (3) to estimate BC storage in the region.

## Materials and Methods

### Site description and sampling

**Fig 1:**
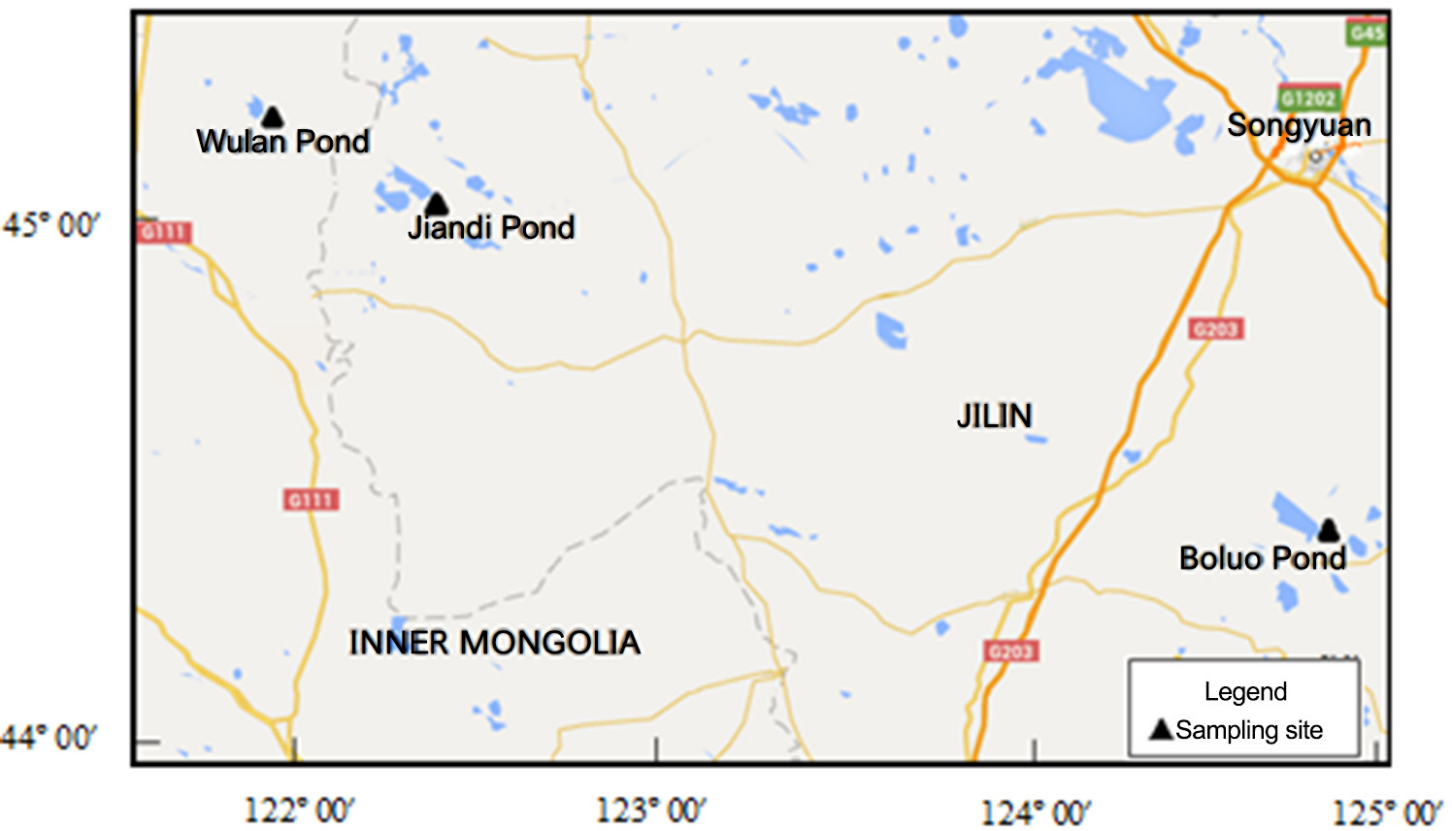
Map of the west part of Songnen Plain, Northeast China and location of the sampling sites in the present study (including: Wulan Pond (WLP), Jiandi Pond (JDP), and Boluo Pond (BLP)). Map from the website of The Gateway to Astronaut Photography of Earth.

The Songnen Plain is located in the northeast of China. The west of the Songnen Plain has a typical semiarid monsoon climate [25]; precipitation decreases from the east (420–460 mm) to the west (350–420 mm), while evaporation increases from the east (1200–1600) to the west (1500–1900 mm) [26]. The wetlands in this study were selected along a gradient of sand desertification from the east at Boluo Pond (BLP) (44°22′49″N, 124°49′12″E) to the west at Wulan Pond (WLP) (45°09′12″N, 121°56′46″E) in March 2005 [25] (Fig 1). In this study, we selected three typical profiles along the gradient, which is the common approach for studying historical changes. The Dabusu profile we previously sampled is located near the lakeshore. The hydrodynamic force of the lake, which could additionally influence the BC, has influenced the profile. For the purpose of study as well as time and cost constraints, we selected three of the four profiles reported in previously study [25]. Sand desertification is relatively a definition based on percentages of the sands and clays. Landforms, climate conditions and other macrocosmic indexes have also been used to distinguish it. In addition to profile analysis, we collected the information on local vegetation types from available documents and historical data on the area. A combination of laboratory analysis and field vegetation data is a common and effective approach to determine different processes and backgrounds of sand desertification [25, 27–28]. The WLP area is covered by natural secondary forest; brushwood vegetation masks the Jiandi Pond (JDP) and reeds dominate the BLP. The Huolin River passes through the region where JDP is nearby and provides an additional water source besides rainfall [29]. The three sites were not inundated when they were sampled. Areas of wetlands in WLP, JDP, and BLP were 76, 230, and 70 km^2^, respectively [25]. Peatland profiles of WLP (56 cm depth), JDP (45°00′05″N, 122°20′09″E, 54 cm depth) and BLP (54 cm depth) were sliced at 2-cm intervals in the field, and individual samples were stored in polyethylene plastic bags and air dried before analysis.

### Methods

#### Black carbon and physiochemical properties

In this study, the dichromate oxidation method [30] modified by Gao et al. (2014) [31] was used to measure the BC contents in the present study. By comparing the effects of different methods on resulting BC contents, Masiello (2004) [17] found that the thermo chemical method could achieve a larger BC fraction than chemical methods due to some charcoals being included. Therefore, the dichromate oxidation method [31] was used to measure BC contents in peat soils that generally contained a large fraction of OM. Specifically, we increased steps of NaOH solution and achieved higher BC contents than those with CTO-375. However, the contents measured with CTO-375 might be lower than those from the benzene-polycarboxylic acids method (BPCAs) due to the interference of humic acid in peatlands.

The Pb contents, like BC, in the soil are usually used as an indicator of the intensity of human activities [32], thus we also analyzed it in the study with ICPS-7500 (Manufactured by Shimadzu, Japan). Other peat soil parameters including grain-size (clay (<4 μm) and sand (>63 μm)), pH and TOC were analyzed in the study; the methods were described in detail by Wang et al. (2006) [25, 33].

#### Chronology

The radiometric dating technique of ^210^Pb was used for dating peat profiles and reconstructing the time frame of the three profiles in this study to correlate to the influences of human activities. The ^210^Pb age was obtained by applying the ^210^Pb data in the three profiles using the constant rate of supply model (CRS) [34], and measured at the State Key Laboratory of Lake Science and Environment, Nanjing Institute of Geography and Limnology, CAS. The sediment accumulation rates were calculated based on ^210^Pb chronology combined with physical properties of sediment, detailed by Begy et al. (2009) [35]. The BC fluxes and organic carbon accumulation rates (OCAR) were calculated by multiplying BC contents and OC contents with sedimentation rates. The Songnen Plain has been intensively influenced by agricultural activities, rapid population growth, large-scale land reclamation, industrialization development, and increased consumption of fossil fuels during different periods, especially after 1950’s [36–40]. In this study, based on sand desertification degree, population development data, and the dating results in three sampling areas [36], we divided the time frame (i.e. profile depth) into three periods: prior to-1900, 1900 to 1950, and post-1950.

#### BC storage

As an important component of the carbon pool, BC storage was estimated with the methods similar to those for estimating OC or carbon stock in soils and calculated by the methods modified from Ma et al. (2014) [41] and Morisada et al. (2004) [42]. In this study, for an individual profile, the BC storage was estimated as the following:

$$BC_{k}=\sum_{i=1}^{k}BC_{i}\times DBD_{i}\times D_{i}\times 10;$$

Where BC_k_ (g m^-2^) represents the BC storage of an individual profile above depth k, BC_i_ (mg g^-1^) represents the BC content in layer i, DBD_i_ (g cm^-3^) is the dry bulk density of layer i, which was obtained by the quotient of dry weight and bulk, and D_i_ (cm) is the thickness of the layer. The method of DBD was as follows:

First, put sample aliquots of profile layers into a known-volume and -weight container, and then weighed it with the analytical balance to obtain the wet weight. After oven drying at 105 for 12h to reach constant weight, dry weights were measured to calculate the dry bulk density.

Based the regional marsh area of 1.12×10^3^ km^2^ [43] and information and results we had, we estimated the BC storage as accurate as we can in this first such study done in the region. With more available data and studies done, accuracy would be improved in the future.

#### Data analyses

The two-way (3 time-periods×3 profiles) ANOVA was used to statistically analyze BC content and BC fluxes with time frames and locations (SPSS 20.0). Origin 9 was used to draw the variation of BC and Pb contents with depth, the historical variation of BC fluxes with pH, OCAR, and silt and sand percentages.

## Results

### pH, Pb, OCAR, clay and sand percentages

**Fig 2:**
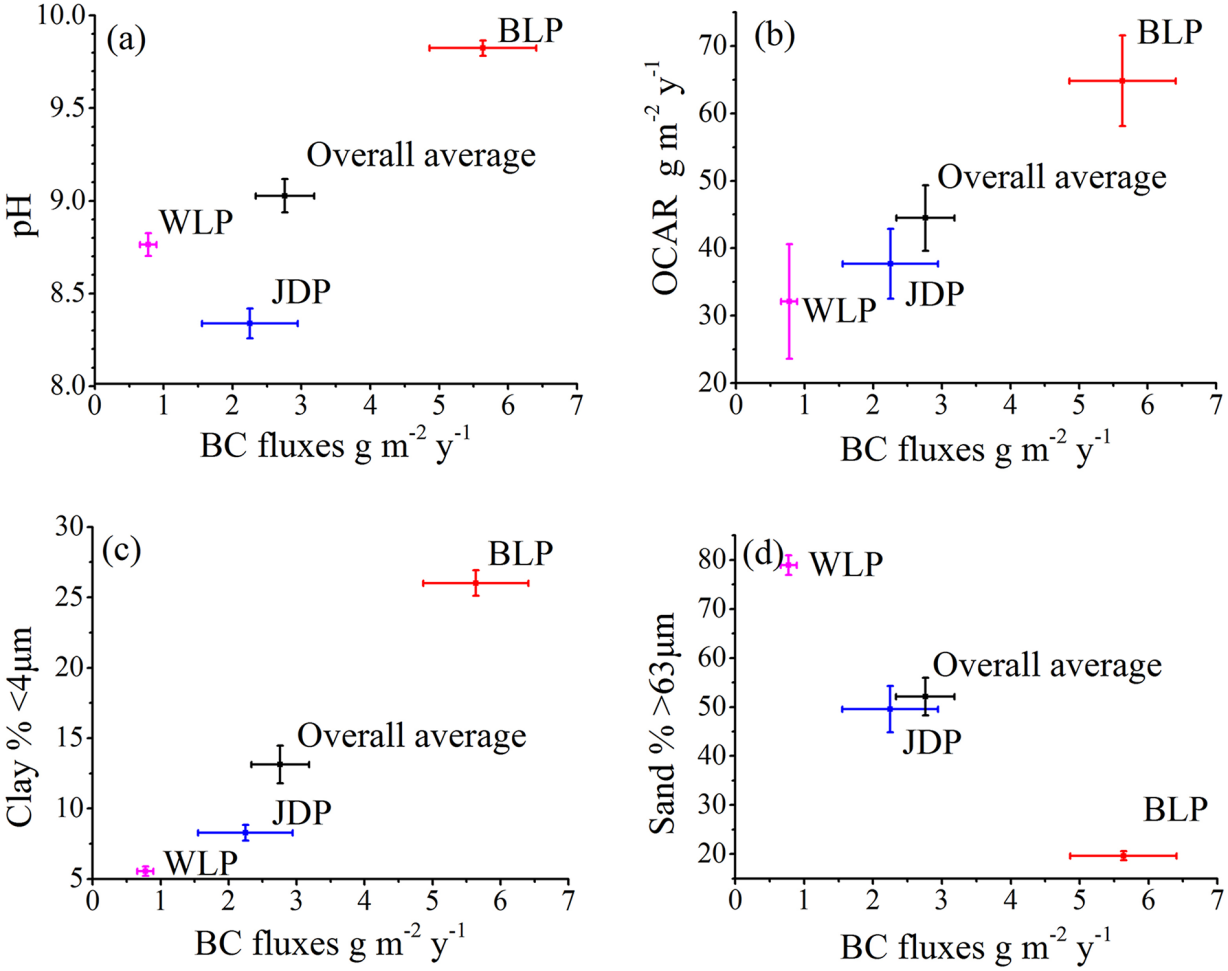
Effect of environmental parameters on BC fluxes in the three profiles of the Songnen Plain (values are expressed as means and standard errors, n≥12). (a) pH, (b) organic carbon accumulation rates, (c) clay percentage, and (d) sand percentage.

In this study, the average soil pH in the profiles was in the order of WLP> BLP> JDP, with an overall mean of 9.03±0.09 (Table 1 and Fig 2), indicating the strong alkaline environment. The Pb contents in the profiles increased with decreasing depth (i.e. from 1850 to the present); the average Pb contents in the three periods were in the order of WLP< JDP<BLP, which is different from the order of pH (Table 1). In addition, the clay percentages in the three profiles varied from 5.55% in WLP to 26.0% in BLP, which had an opposite trend of sand percentages from 78.9% to 19.6% (Table 1). Furthermore, in WLP, JDP and BLP, the average values of OCAR ranged from 32.1±8.5 g m^-2^ y^-1^ in WLP to 64.8±6.7 g m^-2^ y^-1^ in BLP, with an overall mean value of 44.5±4.9 g m^-2^ y^-1^ (Table 1). Unlike the pH and Pb contents, however, OCAR, clay and sand percentages, had no general pattern along the gradient of WLP, JDP and BLP (Fig 2).

**Table 1:** Physiochemical properties, BC storages and C stocks estimated in BLP, JDP and WLP from 1850 to the present (mean ± standard error, n≥12); Pb, BC contents and fluxes in the three profiles during three periods (before 1900, 1900–1950 and after 1950) (mean ± standard error, n≥3).

Sites	WLP			JDP			BLP		
**Periods**	**From 1850 to the present**			**From 1850 to the present**			**From 1850 to the present**		
**pH**	9.82±0.04			8.34±0.08			8.76±0.06		
**clay**	5.55±0.35			8.29±0.56			26.0±0.9		
**sand**	78.9±2.0			49.6±4.7			19.6±0.9		
**OCAR**	32.1±8.5			37.7±5.2			64.8±6.7		
**BC storage**	110.5			565.2			571.4		
**Total carbon storage**	2.4×10^3^			1.0×10^4^			6.5×10^3^		
	**Before 1900**	**1900–1950**	**After 1950**	**Before 1900**	**1900–1950**	**After 1950**	**Before 1900**	**1900–1950**	**After 1950**
**Pb**	12.5±0.2	12.7±0.5	13.7±0.9	13.3±0.4	15.2±1.4	17.1±2.9	18.8±0.9	19.6±1.0	21.6±1.0
**BC**	0.15±0.04^a^	0.14±0.02^ab^	0.36±0.08^c^	0.15±0.01^a^	3.37±1.15^b^	14.9±1.7^c^	1.15±0.23	1.76±0.49	1.46±0.23
**BC fluxes**	0.50±0.15^a^	0.54±0.08^ab^	1.13±0.21^c^	0.17±0.03^a^	2.44±0.93^b^	5.45±0.44^c^	2.99±0.59^a^	5.62±1.67^ab^	7.30±1.06^b^

Units in the table: Clay and sand: %; Pb: μg g^-1^, BC: mg g^-1^; BC fluxes and OCAR: g m^-2^ y^-1^; BC storage and total C stock: g m^-2^. Parameters including pH, clay and sand percentages were quoted from the study of Wang et al. (2008).

Means with different letters of “a, b and c” represent significant difference in LSD test results for BC contents and fluxes in different periods within a profile at a level of p<0.05.

### BC contents, BC fluxes and BC storage

**Fig 3:**
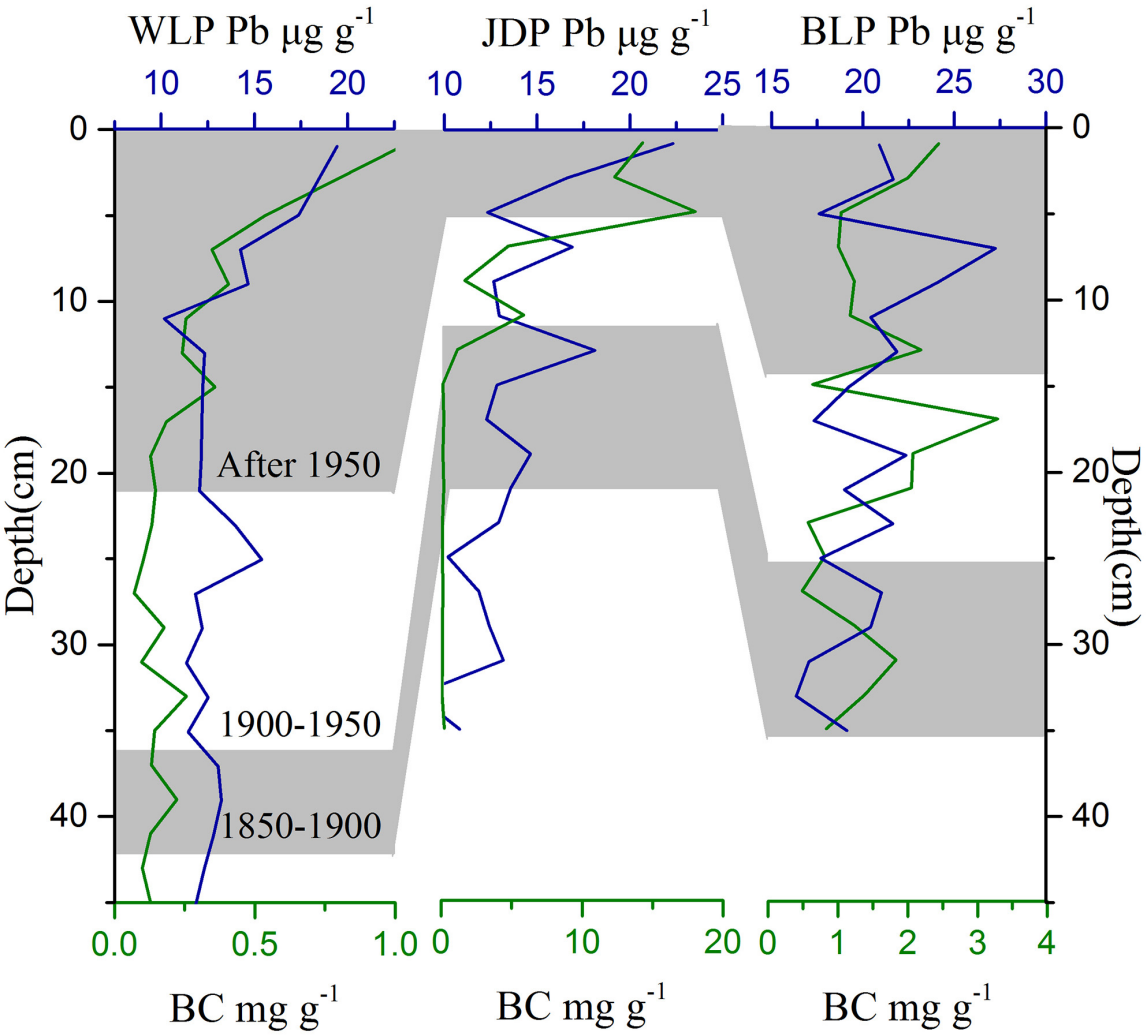
Variation of BC contents (green line) and Pb contents (blue line) with the soil depth in three profiles. Age-depth model was obtained by CRS model and divided into three periods (before 1900, 1900–1950, and after 1950).

The BC contents and fluxes in the three profiles ranged from 0.07 to 18.0 mg g^-1^ and 0.13 to 10.9 g m^-2^ y^-1^, with average values of 1.71±0.48 mg g^-1^ and 2.76±0.42 g m^-2^ y^-1^, respectively (Table 1 and Fig 3). Generally, the BC contents increased with decreasing soil depth (i.e. from 1850 to the present) and almost reached the maximum at the surface soil or near the soil surface for all three profiles. There were significant (*p*<0.001) effects of historical periods and sampling sites on BC contents and BC fluxes and significant interactions between the periods and sites on BC contents (Table 1 and S1 Table). For the JDP profile, BC contents increased significantly between 1900 and 1950 and continued thereafter, while there were no significant differences in BC among the three periods in BLP. The BC storage of an individual profile in the three areas was in the order of WLP (110.5 g m^-2^) < JDP (545.2 g m^-2^) < BLP (572.9 g m^-2^). BC fluxes had a similar trend. The total BC storage in the Songnen Plain was estimated at 4.6×10^11^ g with an average of 410 g m^-2^ (Table 1).

### Correlation analysis to environmental variables

**Fig 4:**
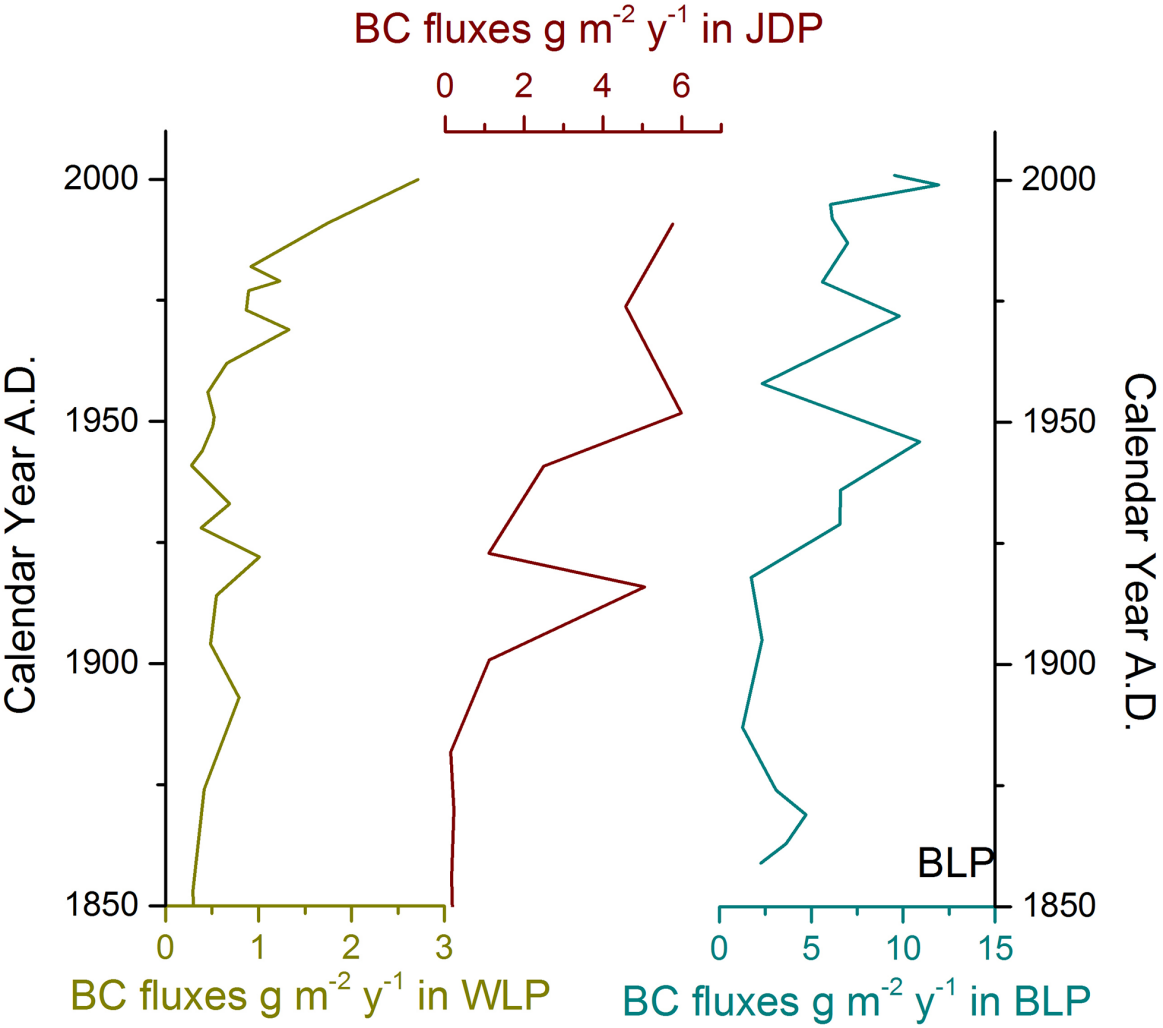
Historical variation of BC fluxes in WLP, JDP, and BLP profiles since 1850.

The BC contents in the WLP and JDP significantly increased in the periods after the 1950s (Fig 3, Table 1, S1 and S2 Tables) compared to those before 1900. The BC contents in the period between 1950 and the present increased by about 2.4, 99.3 and 1.3 folds compared to those before 1900 for WLP, JDP and BLP, respectively. The BC fluxes of the three profiles in this study showed a similar increasing trend with BC contents and were positively related to clay percentages and OCAR (Figs 2 and 4; Table 1 and S1 Table). The average BC fluxes in BLP, JDP and WLP profiles in the period between 1950 and the present significantly increased by the ratios of 1.3, 31.1 and 1.4, respectively, compared to BC fluxes between 1850 and 1900 (Table 1, S1 and S2 Tables).

## Discussion

### Effect of human activities on historical variation of BC fluxes

In this study, we found increasing trends of BC fluxes in our three sampling sites as time passed; especially after the 1950s, BC fluxes had substantially increased. In the period between 1850 and 1900, the BC contents were relatively low and stable in combined with the fact of low human population, thus the contents in this period was treated as the baseline and used to analyze the temporal and spatial changes. A similar increasing trend was reported in Nam Co Lake region (Tibetan Plateau, China), which was attributed primarily to the influence of human activities [44]. Historical archives showed that the Qing government abolished the exit policy in 1851, which attracted the influx of migrants from other provinces [45], causing the population in the region to slowly increase. However, the building of the Middle East Railway in the 1900s, increased regional urbanization and diplomatically opened Northeast China, resulted in a rapid population growth after the 1900’s [39, 46]. Since the 1950s, Northeast China experienced large-scale land reclamation; fire, a quick way of changing natural lands to farmland, was widely used during reclaiming. Population also increased substantially [38]. As a result, rapid and great increases in BC emissions between 1950 and 2000 were reported [47]. For instance, the population density of the nearby city, Keerqin, was 2.40 km^-2^ at the beginning of 1900s, however, increased to 39.72 km^-2^ in 2000. The total population increased from 1.20 million in 1947 to 4.00 million in 2003, with an average growth rate of 4.16% annually [37]. With the increase in population, demand for firewood increased since firewood was the main energy source in rural areas. Every household combusted an average of 5–7×10^6^ g of firewood per year [37] due to the Northeast’s harsh winter, which produced an abundant amount of black carbon [48]. The fossil fuel consumption also greatly rose with population growth. Because of the low percentage usage of gasoline and diesel oil, coal was the primary fuel source, and coal consumption could be used as an indicator of BC produced by fossil fuels in the region, especially in the past decades. For instance, about 35.23 to 37.66 million tons of coal was combusted annually in Jilin Province since 1990 and produced a large amount of BC [40]. Therefore, the BC fluxes in WLP, JDP and BLP would have been greatly influenced by human activities as shown in the previous study in the Tibetan Plateau [48]. In the present study, three periods based on obvious population changes (prior 1900, 1900–1950 and post 1950) coincided with the chronically increase in BC fluxes.

The Pb contents could also be used as an indicator of human activities [32]. Chai et al. (2013) [49] reported a similar trend of increased Pb contents in Yueliang Lake (West of Jilin province, China). The increasing patterns of Pb contents in the three periods (1850–1900, 1900–1950 and post 1950 AD) had the same order of BC contents in the present study. These additional observations indicated that human activities were the key factor affecting the BC fluxes. The BC trend in the present study was similar with the changes of BC for the northern sub-basin in Lake Qinghai [15]; the abrupt increase in char/soot ratios was attributed to the extent of population growth and human activities. Therefore, the BC fluxes changes in the three sampling profiles could be primarily attributed to the increased population in the region in earlier times, as well as large-scale land reclamation (i.e. by using fire) and fossil fuel consumption after the 1950s.

### Effect of sand desertification on spatial variation of BC fluxes

Increasing human population and activities substantially increased BC contents and fluxes, however, environmental changes may additionally affect the BC processes. The soil parameters averaged over soil layers in the three profiles were used to determine whether they influence BC processes. The west of the Songnen Plain had average annual evaporation of 1600 mm and annual precipitation of 350–450 mm [50]; the high evaporation rate resulted in severe alkaline environments and relatively saline marshes. Soil pH was investigated to determine if the pH influenced the BC fluxes because of distinctive differences in pH and severely alkaline environments in the three profiles (average pH>9.0). The average pH in the profile of JDP was lower than that in WLP and BLP (Fig 2(a) and Table 1). This was most likely due to the input of the Huolin River in addition to the rainfall [29]. The pH values might affect types and activities of microorganisms in the soil ecosystems. In general, moderate and neutral pH environments might benefit microbial organic matter decomposition, thus, decreasing OC contents [51]. Previous studies suggested that BC was not a complete inert substance, and could be decomposed further in more favorable environmental conditions [52, 53]. Therefore, like OC, a larger amount of BC would have had been accumulated in a severe alkaline condition (pH>9.0) if unusually high pH could have reduced the microbial decomposition of BC. In the current study, however, the changes of pH were not coincided with BC fluxes in the three profiles (Fig 2(a) and Table 1). With the lowest pH value, BC flux in JDP was not the lowest, but ranked between WLP and BLP. Therefore, the condition of severe alkaline did not play an important role in influencing BC fluxes in the three profiles.

In the current study, there was a great difference in the OCAR among profiles. From the east at BLP to the west at WLP, desertification degree increased and, precipitation decreased (from 420–460 to 350–420 mm) [26]; with more rainfall in the BLP, plants could grow better which led to a higher OCAR, thus likely increasing OC adsorption capacity compared to WLP and JDP (Fig 2(b) and Table 1). In addition, soil accretion between 1900 and the present in WLP, JDP and BLP were about 35, 10 and 25 cm, respectively. Thus, volume dilution effects resulted from both/either higher sand and/or organic matter accumulation should not be neglected in this study. The dilution effects in the three profiles was in the order of WLP>BLP>JDP. Sand deposition likely played an important role for the higher soil accretion of the WLP. Thicker soil depth resulting in a larger soil volume in a given soil surface area and given time could dilute the BC contents more in the WLP and BLP profiles than in the JDP profile. Unlike WLP and JDP being affected greatly by sand desertification, BLP was located outside the sand desertification areas [50], and had the highest clay percentages. Previous studies suggested that the increasing clay percentages could increase the adsorption ability of BC to clay particles in sediments [54–56]. In addition, small clay particles could fill in and clog the pores between sand particles. As a result, high percentages of clay could decrease both oxygen availability and associated aerobic microbial activity, which could then reduce the rates of organic matter decomposition directly and increase the accumulation of organic matter [54]. Therefore, instead of sand deposition, organic matter accumulation with the highest OCAR in the BLP profile could greatly contribute to soil accretion in the BLP profile. Without either fast sand deposition or high organic accumulation with the smallest soil accretion in JDP, BC was concentrated in a small volume of soil in the same soil surface area, thus resulting in a higher BC content in JDP (Fig 3 and Table 1).

Sand desertification in the region is an important environmental variable that may affect BC content and stabilization. The total area of sand desertification in the Songnen Plain had increased substantially and at an alarming rate recently [36]. With more sand in profiles, there could be more oxygen in the pores between sand particles and, as a result, enhance the rate of organic matter decomposition [57]. Direct physical oxidation of BC could be an important mechanism in reducing BC stabilization [53] when larger pores between sand particles allow more air penetration. The lower BC contents and BC fluxes in WLP could be partially correlated to the high percentage of sand, i.e. severe sand desertification. Therefore, sand desertification might be another important factor influencing the BC fluxes. Furthermore, sand desertification decreases the proportion of clays, thus reducing the ability of clays’ adsorption with BC. Percentages of clay and sand could help in estimating the BC storage capacity in the soils. Previous studies suggested that high sand content could reduce the carbon sequestration potential and soil TOC and OM decreased when the abrupt ecosystem changes occurred, such as converting wetland to grassland or arable land [56, 58].

Natural and anthropogenic inputs are two major sources of BC in the region. The incomplete combustion of biomass and fossil fuels is the main anthropogenic source of BC in the three profiles of peatlands. There are apparent differences in three natural conditions in the three profiles in this study: the gradients of precipitation, evaporation, and sand desertification. Precipitation and evaporation resulted in pH and OC differences. The different gradients of sand desertification led to different sand and clay percentages in the three profiles and potentially influenced the BC adsorption ability and sand dilution effect on BC fluxes. The relationships between BC and OCAR, BC and clay percentage, and BC and sand percentages suggest that a wetland with higher OM might have a higher capacity for BC content and fluxes; an ecosystem with a low percentage of sand and high percentage of clay might also have a higher ability for BC content and fluxes.

### BC storage in wetland influenced by sand desertification

The BC storage in WLP, JDP and BLP is shown in Table 1. Based on the regional marsh area of 1.12×10^3^ km^2^ [43], the BC storage in the marsh wetlands of the Songnen Plain was estimated to be about 4.6×10^11^ g, corresponding to approximately 1.7×10^12^ g CO_2_, while the BC produced globally was estimated in a range from 4×10^13^ to 60×10^13^ g per year [12]. As shown in Table 1, the proportion of BC storage in the total C stocks in the region is 6.3%. Therefore, BC storage in this region could play an important role in long-term BC storage and the carbon cycle.

Currently, there is no standard method to measure BC contents, so discrepancies can occur with different techniques for BC analysis. In this study, the dichromate oxidation method [7] modified from Song et al. (2002) [30] was used to measure BC contents because it is potentially more suitable for quantifying BC in peat soils and comparable with other methods like CTO, TOR and BPCAs [30, 59–62]. The average BC content of 1.71 mg g^-1^ in the present study was much lower than that analyzed by the dichromate oxidation method in the peatland of Sanjiang Plain (24.3 mg C g^-1^) [7], but higher than that in Taihu (0.43 to 1.95 mg g^-1^) and Nam Co Lake (0.74 mg g^-1^) [44] measured with the TOR method. However, whether the differences among the studies were due to different regions or methods used will need further investigation. Li et al. (2006) [24] suggested that sand desertification could accelerate vegetation degradation and reduce the contents of SOM. Zhan et al. (2012) [63] indicated that BC contents from different loess zones were in the order of clay loess (0.99±1.07 g kg^-1^)>loess (0.69±0.55 g kg^-1^)>sandy loess (0.37±0.34 g kg^-1^). Previous and current studies suggest that sand desertification could affect BC storage; low sand percentages with high absorption capacity of clays, more oxidation conditions in sand desertification soil, dilution effects of sand and organic accumulation, and degeneration of wetlands as a result of sand desertification in the region might all potentially influence BC storage in wetlands of the western Songnen Plain.

## Conclusions

The relationships between BC and Pb contents with the region’s population growth indicate that human activities are the dominant factor strongly influencing the chronic increases in BC fluxes in the three profiles of the Songnen Plain. The maximum amount of BC fluxes occurred at or near the surface of the three profiles due to increasing amounts of human effects, especially after the 1950s. In addition, sand desertification with high sand, low clay percentages, and fast soil accretion from either sand desertification in some areas or organic accumulation in the others could play roles in BC processes in different profiles; weak absorption by less clay and dilution effects of more sand during sand desertification could decrease the BC contents and BC fluxes. However, the detailed mechanism needs further investigation. Though there has been no standard method for BC measuring, the results of BC in the current study support previous studies in other areas and could potentially contribute to the study on soil carbon storage because this is the first study done on the BC storage in this region.

## Supporting Information

S1 Table Two-way ANOVA and LSD test for BC contents and BC fluxes (total sample numbers = 53). (DOCX)

S2 Table LSD test for BC contents and BC fluxes between sites and periods. Numbers in bold denote significant relationship (p<0.05). (DOCX)
