# Supplementary material for: Temporal and Spatial Changes in Black Carbon Sedimentary Processes in Wetlands of Songnen Plain, Northeast of China
Source: PLoS One. 2015 Oct 15;10(10):e0140834. doi: 10.1371/journal.pone.0140834 (PMC4607433; doi:10.1371/journal.pone.0140834)
Supplement: S1 Table — (DOCX) [file pone.0140834.s001.docx]

**File S1.** **Two-way ANOVA and LSD test for BC contents and BC fluxes (total sample numbers=53).**

|  | **Two-way ANOVA** | | | **LSD** | | | | | |
| --- | --- | --- | --- | --- | --- | --- | --- | --- | --- |
|  |  |  |  | **Sites** | | | **Periods** | | |
|  | Sites | Periods | Interaction of Site  and  Periods | WLP vs JDP | WLP vs BLP | JDP vs BLP | Before 1900  vs  1900-1950 | Before1900  vs  After 1950 | 1900-1950  vs  After1950 |
| **BC contents** | <0.001 | <0.001 | <0.001 | <0.001 | <0.001 | <0.001 | 0.014 | <0.001 | <0.001 |
| **BC fluxes** | <0.001 | <0.001 | 0.053 | 0.026 | <0.001 | <0.001 | 0.051 | <0.001 | 0.008 |

BC contents: mg g^-1^; BC fluxes: g m^-2^ y^-1^.
