# Supplementary material for: Temporal and Spatial Changes in Black Carbon Sedimentary Processes in Wetlands of Songnen Plain, Northeast of China
Source: PLoS One. 2015 Oct 15;10(10):e0140834. doi: 10.1371/journal.pone.0140834 (PMC4607433; doi:10.1371/journal.pone.0140834)
Supplement: S2 Table — (DOCX) [file pone.0140834.s002.docx]

**File S2.** **LSD test for BC contents and BC fluxes between sites and period**s**. Numbers in bold denote significant relationship (p<0.05)**

| Variables | Sites | Periods | | | |
| --- | --- | --- | --- | --- | --- |
|  |  | Before1900  vs  1900-1950 | Before1900  vs  After 1950 | 1900-1950  vs  After1950 |  |
| BC contents | WLP(n=23) | 0.979 | **0.038** | **0.017** | |
|  | JDP(n=12) | **0.034** | **<0.001** | **<0.001** | |
|  | BLP (n=18) | 0.233 | 0.487 | 0.516 | |
| BC  fluxes | WLP(n=23) | 0.729 | **0.016** | **0.017** | |
|  | JDP(n=12) | **0.016** | **<0.001** | **0.007** | |
|  | BLP (n=18) | 0.172 | **0.020** | 0.326 | |

BC contents: mg g^-1^; BC fluxes: g m^-2^ y^-1^.
